# Supplementary material for: A META analysis and systematic review of the effects of exercise interventions on middle-aged and elderly patients with depression
Source: PLoS One. 2025 Jan 9;20(1):e0303594. doi: 10.1371/journal.pone.0303594 (PMC11717248; doi:10.1371/journal.pone.0303594)
Supplement: S1 File — (DOCX) [file pone.0303594.s003.docx]

**Pubmed search algorithm**

#1 "Depression"[Mesh Terms]

#2 "depressive symptoms"[Title/Abstract] OR "depressive symptom"[Title/Abstract] OR "symptom depressive"[Title/Abstract] OR "emotional depression"[Title/Abstract] OR "depression emotional"[Title/Abstract]

#3 #1 OR #2

#4 "Exercise"[Mesh Terms]

#5 "Exercises"[Title/Abstract] OR "physical activity"[Title/Abstract] OR "activities physical"[Title/Abstract] OR "activity physical"[Title/Abstract] OR "physical activities"[Title/Abstract] OR "exercise physical"[Title/Abstract] OR "exercises physical"[Title/Abstract] OR "physical exercise"[Title/Abstract] OR "physical exercises"[Title/Abstract] OR "acute exercise"[Title/Abstract] OR "acute exercises"[Title/Abstract] OR "exercise acute"[Title/Abstract] OR "exercises acute"[Title/Abstract] OR "exercise isometric"[Title/Abstract] OR "exercises isometric"[Title/Abstract] OR "isometric exercises"[Title/Abstract] OR "isometric exercise"[Title/Abstract] OR "exercise aerobic"[Title/Abstract] OR "aerobic exercise"[Title/Abstract] OR "aerobic exercises"[Title/Abstract] OR "exercises aerobic"[Title/Abstract] OR "exercise training"[Title/Abstract] OR "exercise trainings"[Title/Abstract] OR "training exercise"[Title/Abstract] OR (("education"[MeSH Subheading] OR "education"[All Fields] OR "Training"[All Fields] OR "education"[MeSH Terms] OR "train"[All Fields] OR "train s"[All Fields] OR "trained"[All Fields] OR "training s"[All Fields] OR "Trainings"[All Fields] OR "trains"[All Fields]) AND "Exercise"[Title/Abstract])

#6 #4 OR #5

#7 "Aged"[Mesh Terms]

#8 Elderly[Title/Abstract]

#9 #8 OR #9

#10 "randomized controlled trial"[Publication Type] OR "randomized"[Title/Abstract] OR "placebo"[Title/Abstract]

#11 #3 AND #6 AND #9 AND #10

**Web of Science search algorithm**

#1 TS=(Depression OR Depressive Symptoms OR Depressive Symptom OR Symptom, Depressive OR Emotional Depression OR Depression, Emotional) and Preprint Citation Index (Exclude – Database)

#2 Exercise, Acute OR Exercises, Acute OR Exercise, Isometric OR Exercises, Isometric OR Isometric Exercises OR Isometric Exercise OR Exercise, Aerobic OR Aerobic Exercise OR Aerobic Exercises OR Exercises, Aerobic OR Exercise Training OR Exercise Trainings OR Training, Exercise OR Trainings, Exercise) and Preprint Citation Index (Exclude – Database)

#3 TS=(Elderly OR Old OR Senior OR Mature OR Older OR Elderly person OR Aged person OR Senior citizen OR Elderly individual OR Advanced in years OR Geriatric OR Over the hill OR Elderly people OR Older generation OR Senior person) and Preprint Citation Index (Exclude – Database)

#4 TS=(randomized controlled trial OR randomized OR placebo OR RCT ) and Preprint Citation Index (Exclude – Database)

#5 #1 AND #2 AND #3 AND #4 and Preprint Citation Index (Exclude – Database)

**Embase search algorithm**

#1 'depression'/exp OR depression

#2 'depressive symptoms':ab,ti OR 'depressive symptom':ab,ti OR 'symptom, depressive':ab,ti OR 'emotional depression':ab,ti OR 'depression, emotional':ab,ti

#3 #1 OR #2

#4 exercise

#5 'exercises':ab,ti OR 'physical activity':ab,ti OR 'activities, physical':ab,ti OR 'activity, physical':ab,ti OR 'physical activities':ab,ti OR 'exercise, physical':ab,ti OR 'exercises, physical':ab,ti OR 'physical exercise':ab,ti OR 'physical exercises':ab,ti OR 'acute exercise':ab,ti OR 'acute exercises':ab,ti OR 'exercise, acute':ab,ti OR 'exercises, acute':ab,ti OR 'exercise, isometric':ab,ti OR 'exercises, isometric':ab,ti OR 'isometric exercises':ab,ti OR 'isometric exercise':ab,ti OR 'exercise, aerobic':ab,ti OR 'aerobic exercise':ab,ti OR 'aerobic exercises':ab,ti OR 'exercises, aerobic':ab,ti OR 'exercise training':ab,ti OR 'exercise trainings':ab,ti OR 'training, exercise':ab,ti OR 'trainings, exercise':ab,ti

#6 #4 OR #5

#7 'Aged':ab,ti OR 'Elderly':ab,ti OR 'Old':ab,ti OR 'Senior':ab,ti OR 'Mature':ab,ti OR 'Older':ab,ti OR 'Elderly person':ab,ti OR 'Aged person':ab,ti OR 'Senior citizen':ab,ti OR 'Elderly individual':ab,ti OR 'Advanced in years':ab,ti OR 'Geriatric':ab,ti OR 'Over the hill':ab,ti OR 'Elderly people':ab,ti OR 'Older generation':ab,ti OR 'Senior person':ab,ti

#8 'randomized controlled trial':ab,ti OR 'randomized':ab,ti OR 'placebo':ab,ti OR 'rct':ab,ti

#9 #3 AND #6 AND #7 AND #8

**Cochrane Library search algorithm**

#1 Depression

#2 (Depressive Symptoms):ab,ti,kw OR (Depressive Symptom):ab,ti,kw OR (Symptom, Depressive):ab,ti,kw OR (Emotional Depression):ab,ti,kw OR (Depression, Emotional):ab,ti,kw

#3 #1 OR #2

#4 exercise

#5 (Exercises):ab,ti,kw OR (Physical Activity):ab,ti,kw OR (Activities, Physical):ab,ti,kw OR (Activity, Physical):ab,ti,kw OR (Physical Activities):ab,ti,kw OR (Exercise, Physical):ab,ti,kw OR (Exercises, Physical):ab,ti,kw OR (Physical Exercise):ab,ti,kw OR (Physical Exercises):ab,ti,kw OR (Acute Exercise):ab,ti,kw OR (Acute Exercises):ab,ti,kw OR (Exercise, Acute):ab,ti,kw OR (Exercises, Acute):ab,ti,kw OR (Exercise, Isometric):ab,ti,kw OR (Exercises, Isometric):ab,ti,kw OR (Isometric Exercises):ab,ti,kw OR (Isometric Exercise):ab,ti,kw OR (Exercise, Aerobic):ab,ti,kw OR (Aerobic Exercise):ab,ti,kw OR (Aerobic Exercises):ab,ti,kw

#6 #4 OR #5

#7 (Elderly):ab,ti,kw OR (Old):ab,ti,kw OR (Senior):ab,ti,kw OR (Mature):ab,ti,kw OR (Older):ab,ti,kw OR (Elderly person):ab,ti,kw OR (Aged person):ab,ti,kw OR (Senior citizen):ab,ti,kw OR (Elderly individual):ab,ti,kw OR (Advanced in years):ab,ti,kw OR (Geriatric):ab,ti,kw OR (Over the hill):ab,ti,kw OR (Elderly people):ab,ti,kw OR (Older generation):ab,ti,kw OR (Senior person):ab,ti,kw

#8 (randomized controlled trial):ab,ti,kw OR (randomized):ab,ti,kw OR (placebo):ab,ti,kw OR (RCT):ab,ti,kw

#9 #3 AND #6 AND #7 AND #8
