# Supplementary material for: A META analysis and systematic review of the effects of exercise interventions on middle-aged and elderly patients with depression
Source: PLoS One. 2025 Jan 9;20(1):e0303594. doi: 10.1371/journal.pone.0303594 (PMC11717248; doi:10.1371/journal.pone.0303594)
Supplement: S2 File — (PDF) [file pone.0303594.s004.pdf]

	Random sequence generation (selection bias)	Allocation concealment (selection bias)	Blinding of participants and personnel (performance bias)	Blinding of outcome assessment (detection bias)	Incomplete outcome data (attrition bias)	Selective reporting (reporting bias)	Other bias
H Vankova 2014	+	?	−	+	+	?	+
J Sims 2006	+	+	−	+	+	+	+
K J Lee 2023	+	+	−	+	+	+	+
K M Chen 2009	+	+	?	−	+	?	+
L Hu 2017	+	?	−	?	+	?	+
M J Choi 2017	+	?	+	+	+	+	+
M L Chen 2023	+	+	−	−	+	+	+
N A Singh 2001	+	+	−	?	+	?	+
S Agui˜naga 2018	+	?	?	?	+	?	+
S J Liao 2018	+	+	−	+	+	+	+
Y Kai 2016	+	+	−	?	+	+	+
Y S Kim 2018	+	?	−	?	?	?	+
