# Supplementary material for: A META analysis and systematic review of the effects of exercise interventions on middle-aged and elderly patients with depression
Source: PLoS One. 2025 Jan 9;20(1):e0303594. doi: 10.1371/journal.pone.0303594 (PMC11717248; doi:10.1371/journal.pone.0303594)
Supplement: Attachment — Submitted filename: PONE-D-24-06785.docx [file pone.0303594.s005.docx]

The research study conducted a meta-analysis and systematic review to explore the effects of exercise interventions on middle-aged and elderly patients with depression. The investigators extensively searched multiple databases and identified 12 relevant papers involving 994 subjects. The meta-analysis indicated a significant overall effect size of exercise interventions on depressive symptoms in this demographic. However, some heterogeneity among studies was observed.

Q1 The introduction should provide more background information on the prevalence and impact of depression in middle-aged and older adults.

Specify the inclusion and exclusion criteria used during the screening process for selecting papers.

Q2 Describe the characteristics of the included subjects (e.g., age range, gender distribution) in more detail.

Q3 Clarify the primary outcome measures used to assess depressive symptoms in the selected studies.

Q4 Provide a flow diagram illustrating the study selection process, as recommended by PRISMA guidelines.

Q5 Discuss the potential sources of heterogeneity among the included studies in the discussion section.

Q6 Consider conducting subgroup analyses based on exercise intensity, duration, and type.

Address the study's limitations, including potential publication bias and the small number of included papers.

Q7 Elaborate on the implications of the findings and how they contribute to the existing literature.

Q8 Provide recommendations for clinicians or policymakers based on the study's results.

Include a brief description of the statistical methods employed in the meta-analysis.

Specify the software used for data analysis and provide details on the statistical models utilized.

Q9 Consider including a forest plot to present the effect sizes of individual studies visually.

Provide a clear definition of "gentle and balanced exercise series" and explain why it is considered particularly efficacious.

Q10 Justify the choice of moderate exercise sessions lasting 30 to 60 minutes as optimal.

Discuss any potential adverse effects or risks associated with exercise interventions in this population.

Q11 Elaborate on the implications of short-term, medium-term, and long-term exercise interventions regarding effectiveness.

Q12 Discuss possible mechanisms underlying the positive effect of exercise on depressive symptoms.

Q13 Offer suggestions for future research directions based on the study's limitations and gaps in knowledge.

Q14 Proofread the manuscript for grammatical errors and ensure consistency in writing style.
