# Supplementary material for: A META analysis and systematic review of the effects of exercise interventions on middle-aged and elderly patients with depression
Source: PLoS One. 2025 Jan 9;20(1):e0303594. doi: 10.1371/journal.pone.0303594 (PMC11717248; doi:10.1371/journal.pone.0303594)
Supplement: Attachment — Submitted filename: Response to Reviewers.docx [file pone.0303594.s006.docx]

Dear reviewer 1:

First of all, we would like to express our sincere thanks to you for your positive comments on our work as well as your valuable suggestions, while your professional opinions are of great significance for us to improve the paper. The following are the revisions and clarifications we have made in response to your comments:

1. Visual representation of study quality assessment: We have now increased the clarity and efficiency of the presentation of each study's quality assessment by adding collateral documents. Specifically, we have adopted the revised charts of the Cochrane Risk of Bias Tool to visualise the quality scores of each included study.

2. Rationale for the selection of the method for assessing publication bias: We added a section in section 3.4.3 of the manuscript to clarify the rationale for selecting the Egger's test as the method for assessing publication bias, including its applicability, advantages, and considerations compared with other methods.

3. In-depth discussion of future research directions: We expanded the conclusions section with a detailed discussion of future research directions, including exploring specific intervention mechanisms, assessing long-term effects, and analysing differences between groups.

4. Discussion of the impact and management of heterogeneity: our more detailed discussion in the meta-analysis section now provides insights into the sources of observed heterogeneity, its impact on outcomes, and how this heterogeneity can be managed in the future, and in order to further explore the sources of this heterogeneity, we first performed sensitivity analyses, followed by a further discussion in which we specifically highlight the measurement tool differences and menopausal women as two sources of heterogeneity with specific implications for the interpretation of study results.

5. Discussion of consistency and discrepancies with existing literature: In the discussion section, we further explored the consistency or discrepancies of our findings with the existing literature and attempted to explain the potential reasons behind these discrepancies.

6. Discussion of implementation and feasibility of interventions: In the discussion section, we added an analysis of the feasibility of implementing specific interventions and how the findings can be translated into clinical practice or public health policy.

7. recommended references: thank you for the recommended literature, we have carefully researched the literature and cited the work in the appropriate sections based on their relevance and contribution.

8. checking of spelling and punctuation: we have thoroughly checked and corrected spelling and punctuation errors in the manuscript to ensure the accuracy and professionalism of the text.

9. Correction of Grammatical Errors: We have carefully proofread the discussion section and corrected all grammatical errors found to improve the reading fluency of the paper.

Thank you again for your valuable comments. We hope that the above revisions will meet your expectations and make our thesis even better. We look forward to your further guidance and suggestions.

With best wishes.

[ZHENG ZHANG] and co-author team

Dear reviewer 2:

First of all, we would like to express our sincere thanks to you for your positive comments on our work as well as your valuable suggestions, while your professional opinions are of great significance for us to improve the paper. The following are the revisions and clarifications we have made in response to your comments:

Q1: We have added epidemiological information about depression in middle-aged and older adults and its impact on individuals and society in the background section. In addition, we have made the inclusion and exclusion criteria more explicit to ensure rigour and transparency of the study.

Q2: We have added a detailed description of the subjects included in the study, including age range and gender distribution, in the Table 1 section to provide more comprehensive background information.

Q3: We have explicitly listed the primary outcome scales used to assess depressive symptoms in the Table 1 section, including the specific instruments and scales used.

Q4: As you suggested, we have provided a flowchart of the study selection process based on the PRISMA guidelines to increase methodological transparency.

Q5: In the results section, we explored possible sources of inter-study heterogeneity and provided detailed explanations where possible. And heterogeneity was also explored in the discussion section.

Q6: We conducted subgroup analyses in section 3.4.4 to explore the potential influence of aspects of exercise intensity, duration and type on the effect size of treating depression in middle-aged and older adults, and to discuss study limitations and potential publication bias.

Q7: We expanded in the discussion section on the implications of the findings of the study and what they mean for clinical practice, and described how these findings add to the existing literature.

Q8: We have added detailed recommendations for practice based on the study findings in the Discussion section, which we hope will be of some assistance to clinicians or policy makers.

Q9: We have uploaded forest plots in the Results section, including forest plots for each subgroup, to visualise the effect sizes of the studies, and have defined "gentle and balanced exercise series" in detail in the Results section and analysed why it is considered particularly effective.

Q10: We have explained in detail in section 3.4.4.3 the rationale for choosing a moderate intensity exercise session of 30 to 60 minutes per session based on the results and have analysed any potential adverse effects or risks that may exist in the discussion section.

Q11: We have discussed in detail the effects of short, medium and long term exercise interventions and the long term sustainability of these interventions.

Q12: We have analysed the physiological mechanisms by which exercise positively affects depressive symptoms in the discussion section.

Q13: We have added a section on limitations of the study and gaps in the existing knowledge in the conclusion section and we have suggested directions for future research.

Q14: We have carefully proofread the manuscript, corrected grammatical errors and ensured consistency in writing style.

Thank you again for your valuable comments. We hope that the above revisions will meet your expectations and make our thesis even better. We look forward to your further guidance and suggestions.

With best wishes.

[ZHENG ZHANG] and co-author team
